# Biographical Notice of the Late George McClellan, M. D.

**Published:** 1850-01

**Authors:** 


*Biographical Notice of the late George McClellan, M. D.* By S. G. Morton, M. D.—It was remarked by a celebrated historian, that a man cannot speak long of himself without vanity; and the maxim may, perhaps, be extended to those who discourse of their friends. On the present occasion, therefore, I shall study to be brief.

George McClellan was born on the 22d of December, 1796, at Woodstock, in Connecticut. His family, which was highly respectable, was in part of Scotch, in part of English lineage, and afforded several enthusiastic partisans of freedom in the war of Independence.

The subject of these remarks, after receiving the usual academical instruction in his native town, and under the watchful eye of an affectionate father, entered the Sophomore class of Yale College at the age of sixteen years, and in due time received the honors of that venerable Institution.

His collegiate life was marked by singular quickness of perception, readiness in the acquisition of knowledge, and an enthusiastic, but immethodical devotion to his studies. His talent particularly displayed itself in mathematics and the languages: in the former, he showed proficiency; in the latter, his attainments were far above mediocrity. He also manifested a fondness for natural history; and his zeal and success in its cultivation, are favourably recorded in the early numbers of the American Journal of Science, then, as now, edited by the distinguished Professor Silliman.

His collegiate education completed, we next find this young man, in the year 1817, pursuing his medical studies in this city under the direction of the late Dr. Dorsey. Here, again, his restless activity and sleepless vigilance in the pursuit of knowledge, were remarked and admired by all; exciting the surprise of his fellow students, and drawing from older heads, the presage of future distinction. He took his medical degree in 1819, and at once entered on the arduous duties of his adopted vocation.

We do not propose to follow the phases of this gifted mind through its professional cycle of thirty years. To the eye of the casual observer, the path of the physician presents, from day to day and from month to month, the same unvaried uniformity,—a perpetual recurrence of similar scenes and circumstances, of which the busy world feels nothing and knows nothing. But how different is it with him, who, in the struggle of distinction. is alternately cheered by hope and dashed by disappointment—warring with caprice, mingling with disease, and contending with death! These things however, are buried with the dead. They pass into oblivion; or at most, nothing remains of them except a few scattered memorials, which rise like wrecks upon the sea to attract the attention of the beholder.

It is to some of these more prominent events in the life of Dr. McClellan, that I now propose to advert. In so doing, I feel conscious of acting in accordance with his views while living; and "the wishes of the dead, when they can be discovered," observes the younger Pliny, "should be law to an honest mind."

After having given a private course of anatomical lectures, Dr. McClellan conceived the bold idea of founding a new medical school. With him, thought and action were simultaneous; a memorial was addressed by himself and others to the Legislature of Pennsylvania, and a charter was obtained in the winter of 1824, for the Jefferson Medical College.

I venture to assert, from a personal knowledge of the time and circumstances, that no professional innovation was ever more unfavorably received by the physicians of Philadelphia, than this. It had a direct tendency to isolate its author, and certainly influenced his destiny throughout life. It was assumed and asserted that there was not patronage for the support of two schools, and that the new one could only succeed at the expense of its elder rival. And inasmuch as the whole scheme was regarded as a professional heresy, it need not be added that its partisans met with no favour.

Dr. McClellan reasoned differently. He maintained that students would flock to this city in numbers proportioned to the increased facilities for education; and that each institution might be amply supported without any conflict of interest. What has been the result? In place of five hundred students, which was the maximum number before this competition was organized, Philadelphia now annually enrols a thousand; embracing a portion of the genius and talant of every state of the Union.

It is important, however, to observe, that owing to the general disapproval of the plan of a new College, Dr. McClellan met with great difficulty in organizing a medical faculty; and his colleagues were unavoidably chosen from among men greatly inferior in talant to himself. Incongruous elements were thus associated together; dissensions arose, and disunion followed. Yet notwithstanding all these adverse circumstances, Dr. McClellan had the satisfaction, in the year 1836, to welcome no less than 360 pupils into the school he had founded.

Dr. McClellan's lectureship was *Surgery*; and he continued his instructions in this branch until the year 1838, when for reasons unknown to the writer of these pages, the professorships of Jefferson College were all vacated by the decision of the Board of Trustees, and a new organization took place from which Dr. McClellan's name was excluded. This new faculty was composed of men of distinguished attainments. The medical public acquiesced in the change; Jefferson College was received into favour, and collegiate competion was thereby legitimised. So true is the adage that times change, and we change with them: "Tempora mutantur et nos mutamur cum illis."

Dr. McClellan thus lived to experience the proverbial misfortune of most pioneers and discoverers, who sow the seed of which others reap the harvest.

Mortified but not discomfited, by this adverse issue of his cherished plans, D. McClelland immediately conceived the project of a third medical school; and with characteristic buoyancy of spirit and determination of purpose, he went in person, accompanied by a single professional friend, to solicit a charter from the State Legislature. Corporate privileges were in consequence, granted to an institution entitled, "The Medical Department of Pennsylvania College" at Gettysburg, and McClellan, with five associates, of whom the writer was one, commenced the initiatory course of lectures in Philadelphia, in November, 1839.

This institution had an auspicious beginning in a class of nearly one hundred pupils, between which number as a maximum, and eighty as a minimum, it continued under the direction of the same faculty for four consecutive years. Notwithstanding this seeming prosperity, it is due to Dr. McClellan's memory to state, that some injudicious pecuniary arrangements, entered into in the first instance, and in which he had no part, tended to embarrass the institution through the entire period to which we have alluded.

The sinister effect of these arrangements was soon felt by all concerned; and nothing but a mutual sense of honor sustained the faculty, in combined exertion, during four annual courses of lectures, the last of which terminated in the spring of 1843.

Soon after this date, the entire faculty resigned their professorships into the hands of the Trustees. The motive that influenced a part of these gentlemen in taking this step, may be inferred from the preceding statement; other members were influenced, at least in degree, by other considerations to which it is unnecessary here to advert. It may, perhaps, be safely asserted, that Dr. McClellan was the only member of the faculty who reluctantly abandoned this, his last and cherished enterprise. His zeal and enthusiasm could see nothing but success in the future; and he never abandoned the conviction, that further perseverance would have been crowned with commensurate reward.

The remaining portion of Dr. McClellan's life was passed in the active duties of his profession. His final illness was severe, his death sudden. On the morning of the 8th of May, 1847, he assisted in the performence of two surgical operations. He came home soon after noonday, complaining of indigestion, which was quickly followed by symptoms analogous to those of bilious colic. These increased every motion in severity. Medicines at length afforded some mitigation of his suffering, and for a short time, gave promise of relief; but it was presently observed, that as his pain abated, exhaustion and restlessness followed. These symptoms increased towards evening, and at eleven o'clock at night, to the surprise and dismay of his family and friends, the hand of death was evidently upon him. His mind continued clear, but his articulation became hurried and indistinct. At midnight he was pulseless, and soon afterwards fell asleep; and in this state of unconscious tranquillity he died at half past one o'clock the same night.

As a surgeon, Dr. McClellan established for himself a reputation that has become proverbial wherever the healing art is esteemed and fostered. Few men in private practice, in this country, have operated so frequently. His list included almost every capital operation known to surgeons, together with others that were original with himself; and these multiplied efforts of his genius were rewarded with a full share of success.

His almost intuitive perception of disease, led to a promptness of decision and a rapidity of operation that were sometimes regarded as akin to rashness. The sight of blood, however profusely poured out, never dismayed him, because, as he remarked, he knew how to control the bleeding vessels. Every cut of the knife was made with the confidence that could result only from knowledge. In the midst of the severest operations, he continued his conversational remarks with the same coolness as if he had been a mere spectator; whence it happened that differently constituted minds inferred that he was unfeeling, and even cruel.

I cannot, perhaps, offer a better commentary on these points than a memorandum of my own, bearing date of March 8, 1845, which I transcribe *vebatum:*

"The day before yesterday I was present at an operation perfomed by Dr. George McClellan, on a gentleman from Virginia. It consisted in the removal of the whole of the *parotid gland* in a schirrous and much enlarged state, from the left side of the face. Nothing could exceed the combined coolness and skill manifested by Dr. M. throughout this terrible operation, which he has now performed for the eleventh time, and hitherto with remarkable success. On this occasion, however, he was doomed to disappointment, for the patient died last night, about thirty-six hours after the operation. McClellan sent for me in the evening, to see the dying man with him, and we met in the chamber of death between ten and eleven o'clock. I had sometimes heard McClellan spoken of as a heartless surgeon, devoid of feeling or sympathy for those who came under his knife; but I can solemnly aver that I have seldom witnessed more unaffected sorrow than he manifested on this occasion. He walked the room incessantly, and repeatedly clasped my hands in his, while he expressed, in emphatic language, the feelings that preyed upon his mind. He moreover assured me, that he seldom performed one of his severer operations without first asking the blessing of God on his undertaking; and that days and nights of painful anxiety, often preceded those great professional efforts which have justly placed his name on the pinnacle of surgical fame. The gurgling respiration of the unfortunate patient announced the near approach of death, and I withdrew, full of sympathy for the agonized emotions of my friend."

Dr. McClellan's excellent classical education was blended with a continued fondness for literary pursuits, and a lively interest in general science. He read much, but wrote little. He always took up his pen with reluctance; and it was only at the earnest and long continued promptings of his friends, that he at length commenced his "Principles of Surgery." The first printed sheet was placed before him during his brief illness; but he was already too much exhausted to notice its contents. The work however, has been ably edited by his son, and it is now before the world an abiding memorial of the skill and genius of its author.

Novelty in practice is not the test of excellence or superiority in either surgery or medicine. The annals of our profession are full of proofs of the truth of this axiom. Dr. McClellan has made no parade of originality; but he has set forth, with the hand of a master, the multiplied experience of more than a quarter of a century; and this experience was no doubt as extensive as that of any private practitioner among us, during that long period of professional toil. Skill, decision and promptness were in him remarkably conspicuous, and they were combined with a judgment that had become matured in the school of observation and reflection. In the "Principles of Surgery," we find no temporizing treatment, no timid practice; but the positive knowledge of a mind that knew and relied upon its own resources.

Dr. McClellan possessed a sensitive and generous spirit, blended with a confiding manner that strongly marked his intercourse with men. His feelings were quickly excited and warmly expressed at the sense of unkindness or injustice; but there was a magnanimity in his nature that readily forgave an injury. He often regretted the differences into which he was led by the impulsive indiscretion of youth; and emphatically declared, that were it possible to live that part of his life over again, his course should be influenced by greater conciliation and forbearance. In connection with this subject, however it must not be overlooked, that the period between the years 1820 and 1830 was one of peculiar professional disunion; and that Dr. McClellan, in common with many of his medical brethren, was hurried into controversies which, if they can not be forgotten, should at least be remembered with charity.

That Dr. McClellan possessed some of those traits called the "infirmities of genius," we are free to admit: but we may observe, in passing that such infirmities are not, perhaps, more common to genius than to dullness itself. The difference is simply this—that they are conspicuous by contrast in the one, while they are despised in the other.

Dr. McClellan was remarkable for exuberance of thought; and this attribute was responded to by corresponding volubility of speech. In lecturing or in conversation, he was never at a loss for words; yet in spite of this amazing fluency, his ideas manifestly accumulated more rapidly than his tongue could give them utterance. He was communicative and confiding to the last degree, without seeming to be governed by those prudential considerations that habitually influence mere cautious minds.

He was married in the year 1820, to Elizabeth, daughter of the late John H. Brinton, Esq., and five children yet survive him. The eldest, who is one of our colleagues, already holds an enviable position in our common profession. The second son, after graduating with great distinction at West Point, fought throughout the final campaign in Mexico, from Vera Cruz to the capital, thus sharing the glory of the "great Captain' who has won the laurels of Cortezunsullied by crime.—*Transactions of the College of Physicians of Philadelphia.*

